# Acute 5-HT_2C_ Receptor Antagonist SB-242084 Treatment Affects EEG Gamma Band Activity Similarly to Chronic Escitalopram

**DOI:** 10.3389/fphar.2019.01636

**Published:** 2020-01-29

**Authors:** Noémi Papp, Szabolcs Koncz, Diána Kostyalik, Tamás Kitka, Péter Petschner, Szilvia Vas, György Bagdy

**Affiliations:** ^1^ Department of Pharmacodynamics, Semmelweis University, Budapest, Hungary; ^2^ MTA-SE Neuropsychopharmacology and Neurochemistry Research Group, Budapest, Hungary; ^3^ Department of Physiology, Development, and Neuroscience, University of Cambridge, Cambridge, United Kingdom; ^4^ NAP-2-SE New Antidepressant Target Research Group, Budapest, Hungary

**Keywords:** serotonin, 5-HT_2C_ receptor, SSRI antidepressant, gamma rhythm, electroencephalography, sleep-wake cycle, anxiety, depression

## Abstract

Serotonin 2C receptors (5-HT_2C_Rs) are implicated in the pathomechanism and treatment of anxiety and depression. Recently, as a new biomarker of depression, alterations in the gamma power of the electroencephalogram (EEG) have been suggested. Chronic treatment with the selective serotonin reuptake inhibitor (SSRI) antidepressant escitalopram has been shown to cause sleep-wake stage-dependent alterations in gamma power. However, despite the antidepressant potency of 5-HT_2C_R-antagonists, there is no data available regarding the effects of selective 5-HT_2C_R-antagonists on gamma activity. Therefore, we investigate the acute effect of the 5-HT_2C_R-antagonist SB-242084 on gamma power in different vigilance stages when given in monotherapy, or in combination with chronic escitalopram treatment. We administered SB-242084 (1 mg/kg, intraperitoneally) or vehicle to EEG-equipped rats after a 21-day-long pretreatment with escitalopram (10 mg/kg/day, *via* osmotic minipumps) or vehicle. Frontoparietal EEG, electromyogram, and motor activity were recorded during the first 3 h of passive phase, after the administration of SB-242084. Quantitative EEG analysis revealed that acute SB-242084 increased gamma power (30–60 Hz) in light and deep slow-wave sleep, and passive wakefulness. However, in active wakefulness, rapid eye movement sleep, and intermediate stage, no change was observed in gamma power. The profile of the effect of SB-242084 on gamma power was similar to that produced by chronic escitalopram. Moreover, SB-242084 did not alter chronic escitalopram-induced effects on gamma. In conclusion, the similarity in the effect of the 5-HT_2C_R-antagonist and chronic SSRI on gamma power provides further evidence for the therapeutic potential of 5-HT_2C_R-antagonists in the treatment of depression and/or anxiety.

## Introduction

Serotonin (5-hydroxytryptamine, 5-HT) 2C receptors (5-HT_2C_Rs) are widely distributed in the brain, and mediate regulatory effects of 5-HT on anxiety, sleep, hormonal secretion, feeding behavior, locomotor activity, as well as learning and memory processes (Bagdy, 1998; Chagraoui et al., 2016). 5-HT_2C_R dysfunction has been implicated in pathological conditions, like anxiety and depression (Chagraoui et al., 2016; Di Giovanni and De Deurwaerdere, 2016). On the other hand, numerous antidepressants, anxiolytics, and antipsychotics have affinities to the 5-HT_2C_Rs, that may be involved in the therapeutic effects of these drugs (Bagdy et al., 2001; Chagraoui et al., 2016; Di Giovanni and De Deurwaerdere, 2016). Thus, 5-HT_2C_R is a promising pharmacological target in the treatment of several neuropsychiatric disorders (Chagraoui et al., 2016; Di Giovanni and De Deurwaerdere, 2016).

Stimulation of 5-HT_2C_Rs has been described to produce anxiety, and in turn, subtype-selective 5-HT_2C_R-antagonists like SB-242084 exerted marked anxiolytic effects (Dekeyne et al., 2000; Bagdy et al., 2001; Kantor et al., 2005). The selective 5-HT_2C_R-antagonists RS-102221 and SB-242084 produced fast-onset antidepressant-like effects in mice (Opal et al., 2014). Also, 5-HT_2C_R-antagonism potentiated the anxiolytic and antidepressant effects of fluoxetine, a selective serotonin reuptake inhibitor (SSRI), moreover, reduced motor side effects in mice (Demireva et al., 2018). Importantly, the interaction of 5-HT and dopamine (DA) systems has been suggested to contribute to these effects (De Deurwaerdere and Di Giovanni, 2017).

Various parameters on the electroencephalogram (EEG) during both wakefulness and sleep have been described to provide biomarkers of depression and of individualized antidepressant therapy (Steiger and Kimura, 2010). Gamma oscillations are relatively high-frequency (>30 Hz) components of the EEG, that provide important clues about neuronal population dynamics (Buzsaki and Wang, 2012). Gamma oscillations have been associated with sensory and cognitive functions, as well as neural plasticity and memory in animals and humans (Cardin, 2016). On the other hand, abnormal gamma activity has been implicated in several psychiatric conditions (Cardin, 2016). Based on the growing body of evidence suggesting the involvement of gamma in the pathomechanism of depression, alterations in gamma oscillations have recently been proposed as a novel biomarker (or endophenotype) for major depression and even for the follow-up of the antidepressant therapies (Fitzgerald and Watson, 2018). Several antidepressants have been shown to alter gamma activity (Fitzgerald and Watson, 2018). For instance, the SSRI escitalopram has been found to decrease gamma power during rapid eye movement sleep (REMS) in acute treatment, while chronic escitalopram increased gamma power during slow-wave sleep stages (Papp et al., 2018). However, it is still an open question whether the drug-induced alterations of gamma activity in different vigilance stages are indicators or causative mediators of therapeutic action, or signs of side effects (Fitzgerald and Watson, 2018).

According to our knowledge, there is a gap in the literature regarding the effects of selective 5-HT_2C_R-antagonists on gamma oscillations in humans and in rodents. So, here we investigate how gamma power (30–60 Hz) is changing in different vigilance stages (i) by acute administration of the highly selective 5-HT_2C_R-antagonist SB-242084, (ii) by chronic administration of the extensively used SSRI escitalopram, (iii) and by their combination.

## Methods

Male Wistar rats, purchased from the local Animal Facility (Semmelweis University, Budapest, Hungary), were used in the experiments. The animals were kept under controlled environmental conditions (21 ± 1°C, 12/12 h light-dark cycle with lights on at 10 AM), and were provided free access to standard rodent chow and tap water. All housing conditions and animal experiments were performed in accordance with the EU Directive 2010/63/EU and the National Institutes of Health “Principles of Laboratory Animal Care” (NIH Publications No. 85–23, revised 1985), as well as specific national laws (the Hungarian Governmental Regulations on animal studies 40/2013). The experiments were approved by the National Scientific Ethical Committee on Animal Experimentation. All efforts were made to minimize pain and discomfort of the animals.

Electroencephalographic (EEG) and electromyographic (EMG) electrodes were implanted under 2% halothane anesthesia, as described earlier (Jakus et al., 2003). The rats weighed 250–280 g at surgery. For EEG, stainless steel screw electrodes were inserted epidurally over the left frontal cortex (L: 2.0 mm, A: 2.0 mm to bregma), left parietal cortex (L: 2.0 mm, A: 2:0 mm to lambda), and over the cerebellum as a ground electrode. For EMG, a pair of EMG electrodes (stainless steel spring electrodes covered by silicon rubber, Plastics One Inc., Roanoke, VA, USA) was placed into the neck musculature. After recovery (7 days), the rats were attached to the EEG system by a recording cable and an electric swivel fixed above the cages. The animals remained connected throughout the whole study.

For chronic pretreatment, the rats received 10 mg/kg/day escitalopram-oxalate solution (ESC Gedeon Richter Plc., Hungary, dissolved in a solution of 0.3 N HCl in distilled water) or vehicle (VEH; solution of 0.3 N HCl in distilled water) *via* osmotic minipump (2ML4, ALZET, 2.5 μl/h, DURECT Corporation, USA) for 21 days. On the 21st day, the rats received intraperitoneal injections of 1 mg/kg SB-242084 [SB; Tocris, UK, dissolved in a solution of 10% (2-hydroxypropyl)-β-cyclodextrin] or vehicle [veh; solution of 10% (2-hydroxypropyl)-β-cyclodextrin] in a volume of 1 ml/kg body weight. The rats were randomly divided into four groups as follows: VEH+veh (*n =* 6), VEH+SB (*n* = 6), ESC+veh (*n* = 6), and ESC+SB (*n =* 6).

EEG, EMG, and motor activity were recorded for at least 3 h after the injections, starting at light onset. The signals were amplified by analogue filters (Coulburn Lablinc System, USA; filtering below 0.50 Hz and above 100 Hz at 6 dB/octave) and subjected to analogue to digital conversion (MVRD-2200 V, Canopus, Japan) with a sampling rate of 128 Hz. Data were stored on a computer for further processing.

The polygraphic recordings were scored using the automated scoring function of Sleep Sign for Animal (Kissei Comtec America Inc., USA) software for 4-s epochs, followed by visual supervision. Six vigilance stages were distinguished based on conventional criteria (Kantor et al., 2004) as follows (see **Supplementary Figure 1** for representative traces). In active wakefulness (AW), the EEG is characterized by low-amplitude activity at beta (14–29 Hz) and alpha (10–13 Hz) frequencies, in addition to intense EMG and motor activity. In passive wakefulness (PW), the EEG pattern is similar to AW, accompanied by a relatively high EMG activity and minimal or no motor activity. In light slow-wave sleep (SWS-1), the EEG is characterized by high-amplitude slow cortical waves (0.5–4 Hz) interrupted by spindles (6–15 Hz), accompanied by reduced EMG activity and no motor activity. In deep slow-wave sleep (SWS-2), the EEG is dominated by continuous high-amplitude slow cortical waves with reduced EMG and no motor activity. In intermediate stage of sleep (IS), that occurs mostly before or after REMS, the EEG is characterized by an association of high-amplitude spindles and theta (5–9 Hz) waves. In REMS, the EEG is dominated by low-amplitude and high-frequency activity with regular theta waves, in addition to the silent EMG and motor activity with occasional muscle contraction (twitching). Epochs that were contaminated with artefacts or contained transition between vigilance stages were discarded.

The quantitative EEG (qEEG) analysis was computed for consecutive 4-s epochs in the frequency range of 0.5–60 Hz by means of fast Fourier transformation (Hanning window, frequency resolution of 0.25 Hz). Adjacent 0.25-Hz bins were summed into 1-Hz bins which are marked by their upper limits. The power values of epochs in AW, PW, SWS-1, SWS-2, IS, and REMS were separately averaged in the summarized 3 h, or in the first, second, and third h respectively, after treatments to obtain power density values for these sleep-wake stages. In this report, we focused on the 30–60 Hz frequency range of the EEG power spectra. Data within 49–51 Hz were excluded from the analysis to avoid contamination of the 50 Hz interference noise from the electrical network. All data were log-transformed before analysis.

For power spectral analysis, comparisons among groups were performed by two-way ANOVA on repeated measures with two main factors: treatment (non-repeated) and gamma frequency bins (repeated), followed by Bonferroni *post hoc* test. To investigate treatment effects in each h, summarized 30–60 Hz power data (excluding those of 49–51 Hz) were analyzed using one-way ANOVA, regarding AW, PW, SWS-1, SWS-2, IS, and REMS respectively. Following detection of a significant main treatment effect, *post hoc* Newman–Keuls tests were conducted. Statistical significance was accepted when *p* < 0.05.

In our experimental design, control rats (VEH+veh) were exposed to the same procedures as drug-treated ones. Thus, the comparison of the drug-treated groups and the control group is aimed at minimizing the distress caused by the injections and the presence of the minipumps, allowing to study the effects of the drugs. For spectral graphs, in favor of better visibility (as EEG power data follow a decreasing tendency with increasing frequencies), we normalized power values of drug-treated rats to the mean power spectral values of the control ones (VEH+veh group), thus showing relative data. Absolute data are presented on the bar graphs, as summarized power values. Data are expressed as mean ± SEM of six animals per group.

## Results

**Figure 1:**
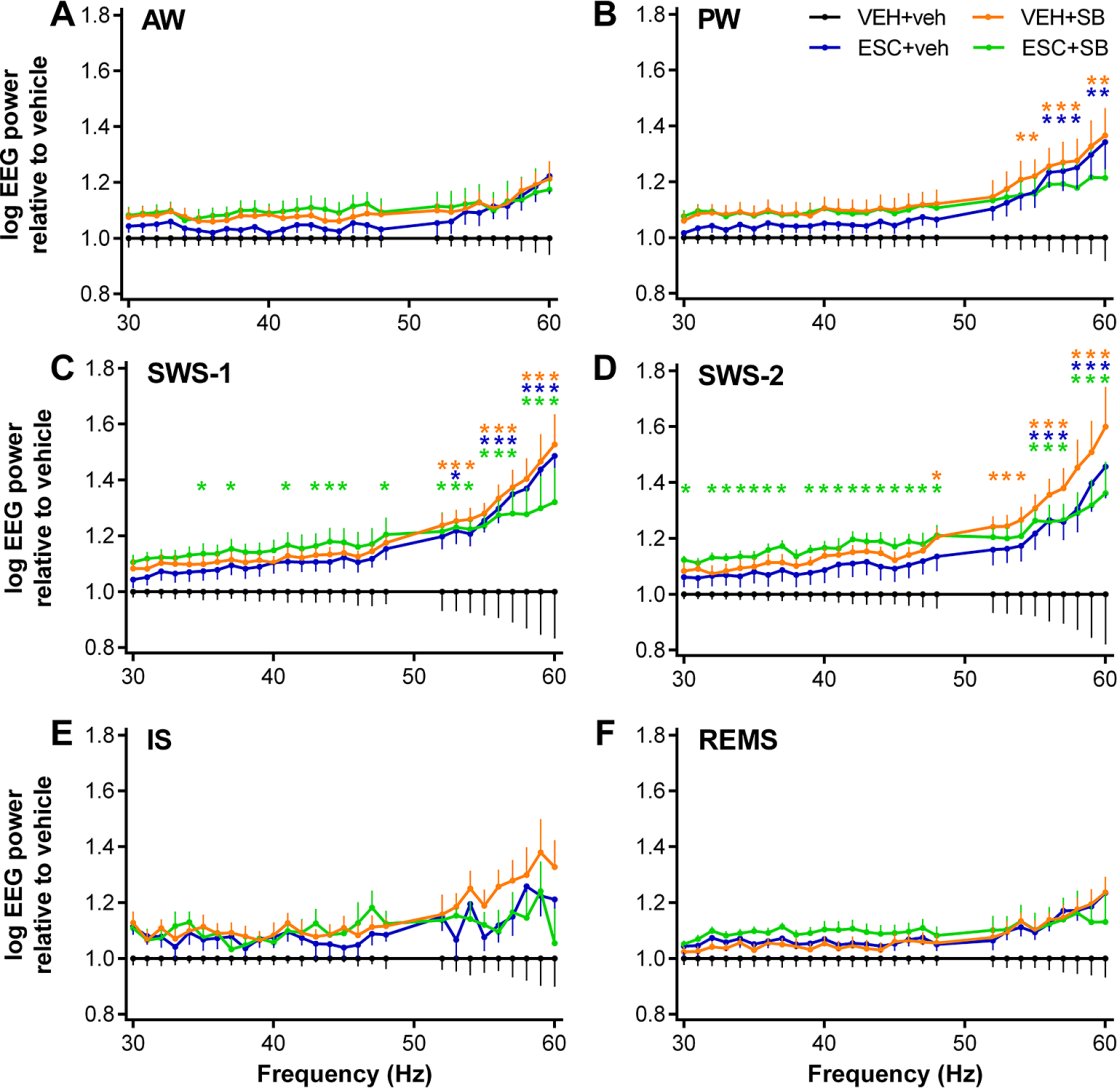
EEG power changes in the gamma frequency range (30–60 Hz) following the administration of acute SB-242084 (VEH+SB), chronic escitalopram (ESC+veh), and their combination (ESC+SB) in the first 3 h of the passive phase. Power spectra are shown in the following six vigilance stages: **(A)** active (AW) and **(B)** passive wakefulness (PW), **(C)** light (SWS-1) and **(D)** deep slow-wave sleep (SWS-2), **(E)** intermediate stage (IS), and **(F)** rapid eye movement sleep (REMS). EEG power data are presented as mean ± SEM, relative to the VEH+veh control group (n = 6 animals per group). *Significant *post hoc* results compared to control, *p* < 0.05.

Effects of the drugs (acute SB-242084, chronic escitalopram, and their combination) on the qEEG in the 30–60 Hz gamma range were evaluated separately for the six vigilance stages by averaging the power data in the first 3 h of the passive phase (**Figure 1**). The analysis revealed that all of the drugs caused vigilance stage-dependent alterations in this frequency range, with a tendency for elevation in each stage, compared to the VEH+veh group.

During AW, ANOVA results showed no significant alterations (treatment effect: *F*_3,20_ = 1.917, *p* = 0.1594; treatment x frequency interaction: *F*_81,540_ = 1.218, *p* = 0.1081; **Figure 1A**). However, during PW, a moderately elevated power line was seen in the SB groups, and at the higher frequencies also in the ESC+veh group (treatment effect: *F*_3,20_ = 2.217, *p* = 0.1176; significant treatment x frequency interaction: *F*_81,540_ = 2.519, *p* < 0.001; **Figure 1B**).

The most prominent effects were observed during slow-wave sleep stages, where the acute SB-242084, the chronic escitalopram, and also the combined treatment caused a power elevation. This was supported by significant ANOVA results in SWS-1 (treatment effect: *F*_3,20_ = 3.692, *p* = 0.0290; significant treatment x frequency interaction: *F*_81,540_ = 2.693, *p* < 0.001; **Figure 1C**), and in SWS-2 (significant treatment effect: *F*_3,20_ = 4.787, *p* = 0.0113; significant treatment x frequency interaction: *F*_81,540_ = 1.911, *p* < 0.001; **Figure 1D**).

The gamma power increase was not significant during IS (treatment effect: *F*_3,20_ = 2.349, *p* = 0.1031, treatment x frequency interaction: *F*_81,540_ = 1.241, *p* = 0.0878; **Figure 1E**). Finally, during REMS, neither the acute SB-242084 nor the chronic escitalopram affected the EEG power in this frequency range (treatment effect: *F*_3,19_ = 2.515, *p* = 0.0891; treatment x frequency interaction: *F*_81,513_ = 1.226, *p* = 0.1014; **Figure 1F**). We note that the data of only n = 5 animals were used in the ESC+SB group, as one animal did not spend any time in REMS in this time interval.

For significant Bonferroni *post hoc* differences following the significant two-way ANOVA results described above, see **Figure 1** for each vigilance stage. No significant *post hoc* differences were seen in any of the stages regarding comparisons of VEH+SB vs. ESC+veh, VEH+SB vs. ESC+SB, and ESC+veh vs. ESC+SB, respectively.

**Figure 2:**
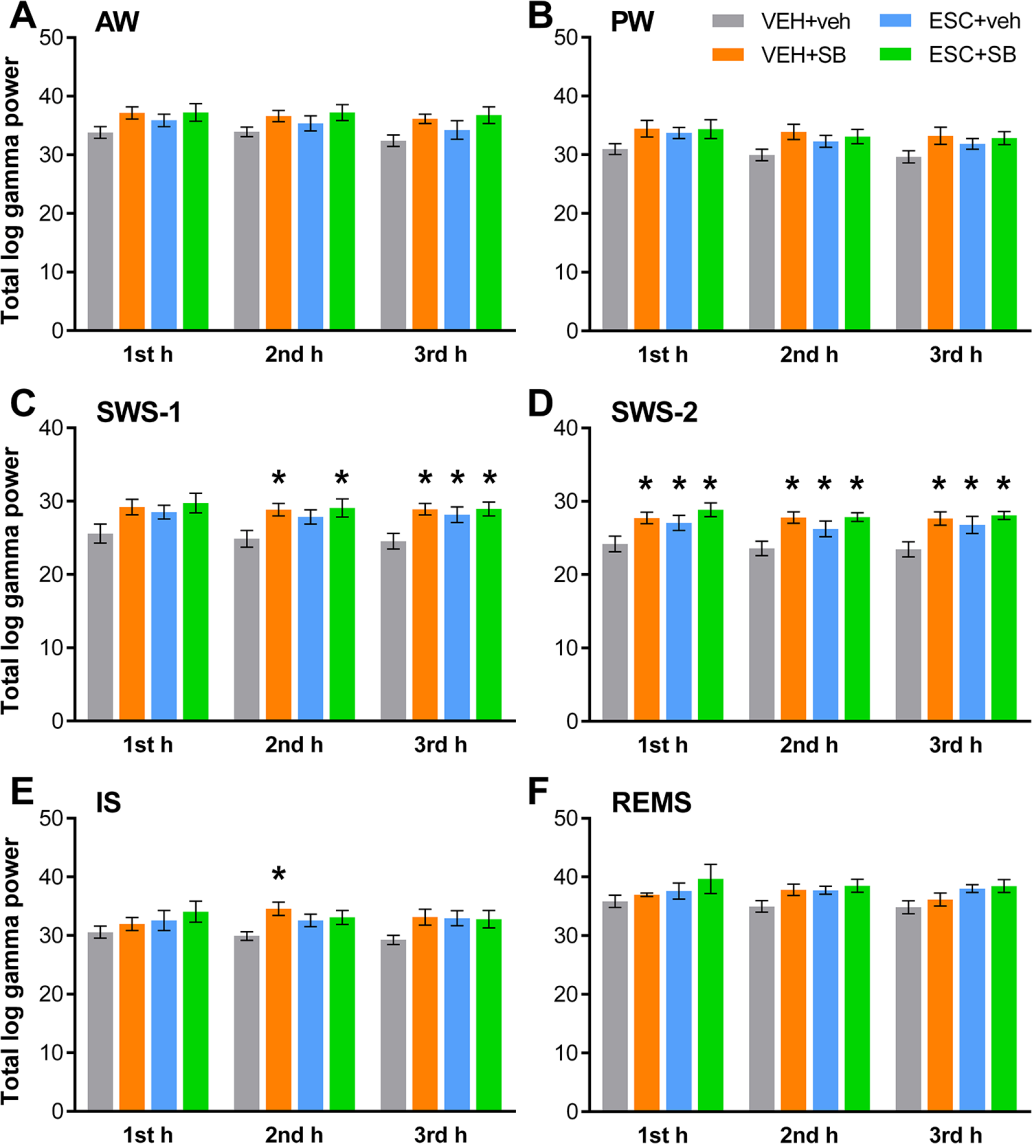
Total EEG power in the gamma frequency range (30–60 Hz) following the administration of acute SB-242084 (VEH+SB), chronic escitalopram (ESC+veh), and their combination (ESC+SB) in the first, second, and third h of the passive phase, during **(A)** active (AW) and **(B)** passive wakefulness (PW), **(C)** light (SWS-1) and **(D)** deep slow-wave sleep (SWS-2), **(E)** intermediate stage (IS), and **(F)** rapid eye movement sleep (REMS). Summarized EEG power data are presented as mean ± SEM (of n = 6 animals per group). *Significant *post hoc* results compared to the control group (VEH+veh), *p* < 0.05.

The sleep-wake stage-dependent effects of the drugs on total gamma power were analyzed hourly. One-way ANOVA revealed that although in the first h of SWS-1, the treatment effect was not significant yet (*F*_3,20_ = 2.544, *p* = 0.0851), in the second (*F*_3,20_ = 3.331, *p* = 0.0403) and third h (*F*_3,20_ = 4.607, *p* = 0.0131) the power elevations were significant. In SWS-2, ANOVA showed significant treatment effects in each h (*F*_3,18_ = 4.062, *p* = 0.0228; *F*_3.20_ = 5.348, *p* = 0,0072; *F*_3,20_ = 4.910, *p* = 0.0102, respectively). Also, a significant effect was found in the second h of IS (*F*_3,20_ = 3.452, *p* = 0.0360), but not in the first or third h. Finally, no significant treatment effect was found in these hours in AW, PW, and REMS. For significant *post hoc* results, see **Figure 2** for each vigilance stage.

## Discussion

This is the first study investigating the acute effects of the selective 5-HT_2C_R-antagonist SB-242084 on the gamma frequency band of the EEG spectra in different vigilance stages. In addition, it was tested with or without chronic SSRI pretreatment. Here we report an enhanced gamma band power during SWS-2, SWS-1, and PW following administration of acute SB-242084, (1 mg/kg, ip.), in rats. Our findings about the additional 21-day-long pretreatment with escitalopram (10 mg/kg/day, osmotic minipumps) demonstrate that SB-242084 did not modify further the gamma power altered by the chronic antidepressant treatment. Moreover, the acute effects of SB-242084 and the chronic effects of escitalopram on gamma power seemed very similar in most vigilance stages. However, the effects of the combination of these two treatments were not considered as additive.

Brain functions related to gamma activity were discussed in the introduction section. Gamma oscillations have been described in multiple areas of the brain, such as neocortex, entorhinal cortex, hippocampus, thalamus, amygdala, olfactory bulb, and striatum. Their generation is tied to perisomatic inhibition; most of all, the activity of parvalbumin-positive interneurons *via* gamma-aminobutyric acid (GABA)_A_ synapses (Buzsaki and Wang, 2012). Gamma oscillations are thought to play an important role in depression, which is supported by both human and animal findings. Namely, gamma abnormalities were measured in depressed patients, and also in animal models of depression, where changes in gamma were associated with therapeutic recovery (Khalid et al., 2016; Noda et al., 2017; Fitzgerald and Watson, 2018).

Various pharmacological (targeting serotonin, noradrenaline, dopamine, and glutamate systems) and non-pharmacological treatments of depression have been shown to alter gamma activity (Fitzgerald and Watson, 2018). For instance, escitalopram, the extensively used SSRI antidepressant, acting on the serotonergic system, has been shown to reduce gamma power during REMS by acute treatment, while its chronic administration increased gamma power in slow-wave sleep stages, in rats (Papp et al., 2018). Importantly, antidepressants acting on the noradrenergic system, such as reboxetine and desipramine, increased gamma (as well as theta) power in the septo-hippocampal system following acute treatment, whereas the SSRI fluvoxamine failed to produce such an effect in the same study (Hajos et al., 2003a). The putative next-generation antidepressants, such as ketamine and its metabolite (2R,6R)-hydroxynorketamine have been shown to prominently boost gamma power when applied in antidepressant-relevant doses. These effects and also their antidepressants-like actions were prevented by pretreatment with an α-amino-3-hydroxy-5-methyl-4-isoxazole propionic acid receptor (AMPAR) inhibitor (Zanos et al., 2016). Finally, Fitzgerald and Watson have recently proposed that gamma signaling may provide a biomarker of the antidepressant effects of all classes of antidepressants, as, according to their hypothesis, all classes of antidepressants may have common final effects on the limbic circuitry (Fitzgerald and Watson, 2019).

In this study, we examined drugs acting on the 5-HT system, namely, an antagonist of 5-HT_2C_R and an SSRI with the main action of increasing the extracellular level of 5-HT by inhibiting its synaptic reuptake *via* the 5-HT-transporter. A growing body of evidence suggests that 5-HT_2C_R-antagonism might provide beneficial effects in the therapy of depression and anxiety, although these effects of the selective 5-HT_2C_R-antagonists have not been tested in clinical studies yet. Supporting the relevance of combining 5-HT_2C_R-antagonists with SSRIs, the acute co-administration of the selective 5-HT_2C_R-antagonists SB-242084 or RS-102221 with the SSRI citalopram augmented the citalopram-induced elevations of 5-HT levels in regions implicated in the pathophysiology of depressive disorders, in a microdialysis study in rats (Cremers et al., 2004). Pretreatment with SB-242084 also reversed the acute citalopram-induced enhancement of fear expression in rats (Burghardt et al., 2007). Furthermore, a recent paper has shown that acute SB-242084 treatment after chronic (21 days) fluoxetine treatment reduced the anxiety- and depression-like behaviors, and ameliorated the SSRI-induced side effects in mice (Demireva et al., 2018).

In the present study, 1 mg/kg SB-242084 dose was chosen, based on former behavioral (Kennett et al., 1997; Martin et al., 2002; Opal et al., 2014) and sleep and/or EEG (Popa et al., 2005; Sorman et al., 2011; Bogathy et al., 2019) studies in rodents. Importantly, Kantor et al. have demonstrated that although 0.3 and 1.0 mg/kg doses of the drug produced similar anxiolytic effects, only the higher dose affected the sleep-wake architecture prominently (wake-promoting effect with reduction of SWS-2), in Sprague-Dawley rats (Kantor et al., 2005). A REMS-reducing effect have been found at 1 mg/kg dose in Wistar rats, and theta power elevations during AW and REMS have also been observed following this treatment (Kostyalik et al., 2014).

The effect of the SB-242084 and escitalopram on gamma activity was analyzed separately in vigilance stages, since brain functions and associated oscillatory activity, and neurotransmitter levels show characteristic differences between stages. The role of gamma oscillations in functions linked to wakefulness such as focused attention, cognition, and sensory processing is well documented (Cardin, 2016), however, their functional importance during sleep is less understood. During REMS, a role of gamma (beyond theta) oscillations in memory consolidation has been suggested (Montgomery et al., 2008). A recent paper has proposed that increased gamma power in phasic REMS microstate in certain areas is associated with emotional processing, in humans (Simor et al., 2019). During non-REMS, gamma oscillations might be associated with phasic increases of neural activity during slow oscillations (Valderrama et al., 2012). A microelectrode-study in monkeys and humans has further emphasized the role of gamma (and beta) oscillations during non-REMS, namely, a high oscillation coherence suggests a contribution a contribution of gamma to memory consolidation to memory consolidation during non-REMS (Quyen et al., 2016).

We found that a single dose of SB-242084 elevated gamma power during SWS-2, SWS-1, and— to a lesser extent— PW, but not during REMS, IS, and AW.

5-HT neurons fire most actively during wakefulness, their activity decreases during slow-wave sleep, and falls silent during REMS. The 5-HT system has thus been considered a key modulator of sleep-wake behavior (Monti, 2011) and brain oscillations, although the cellular mechanisms and the receptors involved in these processes are complex and poorly understood (Puig and Gener, 2015). 5-HT *per se* has been found to affect gamma activity in the rat prefrontal cortex through 5-HT_1A_Rs and 5-HT_2A_Rs, but not 5-HT_2C_Rs, resulting in an overall decrease in amplitude in anesthetized rats (Puig et al., 2010). Furthermore, there is a mounting evidence supporting the importance of the DA system in synchronization of fast-spiking interneurons, thus generating/regulating gamma oscillations (Furth et al., 2013).

5-HT_2C_R is presumably the 5-HT receptor subtype with the most widespread distribution in the brain. It has been described on GABAergic interneurons in numerous regions, and is also expressed by DA neurons. However, this receptor subtype is not expressed by 5-HT and noradrenergic (NA) neurons. The role of 5-HT_2C_Rs in the modulation of the monoaminergic, and intriguingly, the DA systems has been underlined by several preclinical studies (Di Giovanni and De Deurwaerdere, 2016; De Deurwaerdere and Di Giovanni, 2017). Local stimulation of the 5-HT_2C_Rs in specific brain areas produced anxiety in a region-dependent manner, and in turn, subtype-selective 5-HT_2C_R-antagonists like SB-242084 exerted marked anxiolytic actions (Bagdy et al., 2001; Di Giovanni and De Deurwaerdere, 2016). Several anxiolytics and antidepressants possess 5-HT_2C_R antagonist properties, for example, numerous tricyclic antidepressants, mianserin, mirtazapine, trazodone, nefazodone, fluoxetine, and agomelatine (Martin et al., 2014). On the other hand, selective 5-HT_2C_R antagonists have been suggested as putative fast-onset antidepressants based on preclinical studies (Opal et al., 2014).

A few studies have been published regarding qualitative (affecting sleep-wake architecture) and quantitative (affecting power spectra, i.e. oscillations) sleep-wake effects of 5-HT_2C_R antagonists, and SB-242084 particularly. Kantor et al. have investigated the dose-dependent effects of SB-242084 on anxiety, vigilance, and EEG spectra below 30 Hz in rats. They have found increased theta power in wakefulness (that suggests a possible a possible cognitive-enhancing effect, as theta activity is associated with learning and memory processes) even at 0.1 mg/kg dose. In contrast, the drug increased wake and suppressed SWS-2 in the first h of passive phase only at 1 mg/kg dose. Regarding delta, alpha, or beta frequency bands during wakefulness, REMS, SWS-1, or SWS-2, no significant alteration has been reported in this paper (Kantor et al., 2005). Kostyalik et al. have described that SB-242084 elevated PW duration, and suppressed REMS which effect was attenuated by chronic ESC pretreatment, in rats. Regarding qEEG, they have found enhanced theta power during AW and also during REMS that was not further modified by chronic escitalopram pretreatment (Kostyalik et al., 2014). The theta-enhancing effect of the 5-HT_2C_R-antagonist and thus the role of 5-HT_2C_R in the regulation of theta activity have been supported by Hajos et al. (2003b).

The effects of escitalopram and other SSRIs on sleep-wake architecture and EEG parameters in humans and rodents are more extensively studied (Wilson and Argyropoulos, 2005; Vas et al., 2013; Fitzgerald and Watson, 2019). Recently, we have reported gamma power alterations following acute (reduction during REMS) and chronic (elevation during SWS-1 and SWS-2) administration to rats (Papp et al., 2018).

Interestingly, the acute effects of SB-242084 were similar to those caused by chronic escitalopram, with other words, selective antagonism on 5-HT_2C_R produced similar effects on gamma activity as the chronic blockade of the 5-HT-transporter and additional effects escitalopram exerts. In that case, we presume that effects of 5-HT, namely, the desensitization of 5-HT_1A_R (and possibly also 5-HT_2A_R) may account for the increase in gamma power in those stages, as we have proposed in our previous paper (Papp et al., 2018).

To explain our findings, we must take into consideration that 5-HT_2C_Rs can modulate 5-HT, NA, and importantly, DA neuronal activity (Di Giovanni and De Deurwaerdere, 2016). Constitutive activity of the 5-HT_2C_Rs has been reported to exert inhibitory control on DA neuron activity, which was reversed by systemic administration of 5-HT_2C_R antagonists. SB-242084 and SB-206553 significantly enhanced basal DA release in DA innervated areas of the rat brain. However, the SB-242084 elicited DA release was small, and reached a maximum compared with SB-206553 (De Deurwaerdere et al., 2004). Interestingly, SSRIs have also been implicated in regulating DAergic signaling (that might indicate a possible common feature of the two investigated drugs), although the available literature data are ambiguous. While acute escitalopram has been reported to increase the firing rate and bursting of neurons in the ventral tegmental area (Schilstrom et al., 2011), another study has demonstrated that a 2-week-long administration of escitalopram decreased the firing rate and bursting of these neurons, in anesthetized rats (Dremencov et al., 2009).

The findings of this study should be interpreted in the context of some limitations. SB-242084 was administered in one dose only, and we did not test whether the gamma-enhancing effect of the drug is dose-dependent. In addition, our analysis was limited to a maximum of 60 Hz due to the Nyquist limit determined by the nominal sampling rate, thus further work is required to investigate faster gamma frequencies, as well as high-frequency oscillations (HFO) and ripples.

Taken together, our findings show similar effects between acute SB-242084 and chronic escitalopram on gamma activity, which support preclinical (and indirect clinical) evidences that acute or short-term treatment with 5-HT_2C_R-antagonists may have antidepressant effects. Thus, our work might provide further evidence that 5-HT_2C_R-antagonists may be beneficial in the treatment of depression and/or anxiety. Whether alterations of gamma activity are markers of therapeutic drug action or mediators of therapeutic effect, remains an unresolved question that needs further studies.

## Data Availability Statement

The datasets generated and analyzed during the current study are not publicly available due to ongoing analysis for future publication, but are available from the corresponding author upon reasonable request.

## Ethics Statement

All housing conditions and animal experiments were performed in accordance with the EU Directive 2010/63/EU and the National Institutes of Health “Principles of Laboratory Animal Care” (NIH Publications No. 85-23, revised 1985), as well as specific national laws (the Hungarian Governmental Regulations on animal studies 40/2013). The experiments were approved by the National Scientific Ethical Committee on Animal Experimentation, and permitted by the government (Food Chain Safety and Animal Health Directorate of the Central Agricultural Office, Permit No. 22.1/1375/7/2010).

## Author Contributions

GB and TK designed the experiments. DK, SV, and PP performed the experimental procedures. GB, DK, SV, and NP contributed to data analysis. NP, SK, SV, and GB interpreted the results. NP, SK, and GB wrote the first draft of the manuscript. All authors have read and approved the final manuscript.

## Funding

This work was supported by the National Development Agency (KTIA_NAP_13-1-2013-0001) Hungarian Brain Research Program (Grant No. KTIA_13_NAP-A-II/14), NAP 2.0 (Grant No. 2017-1.2.1-NKP-2017-00002); ITM/NKFIH Thematic Excellence Programme, Semmelweis University; and the SE-Neurology FIKP grant of EMMI.

## Conflict of Interest

The authors declare that the research was conducted in the absence of any commercial or financial relationships that could be construed as a potential conflict of interest.
