# Supplementary material for: Acute 5-HT2C Receptor Antagonist SB-242084 Treatment Affects EEG Gamma Band Activity Similarly to Chronic Escitalopram
Source: Front Pharmacol. 2020 Jan 29;10:1636. doi: 10.3389/fphar.2019.01636 (PMC7000428; doi:10.3389/fphar.2019.01636)
Supplement: Supplementary file 1 [file Image_1.pdf]

Supplementary Material

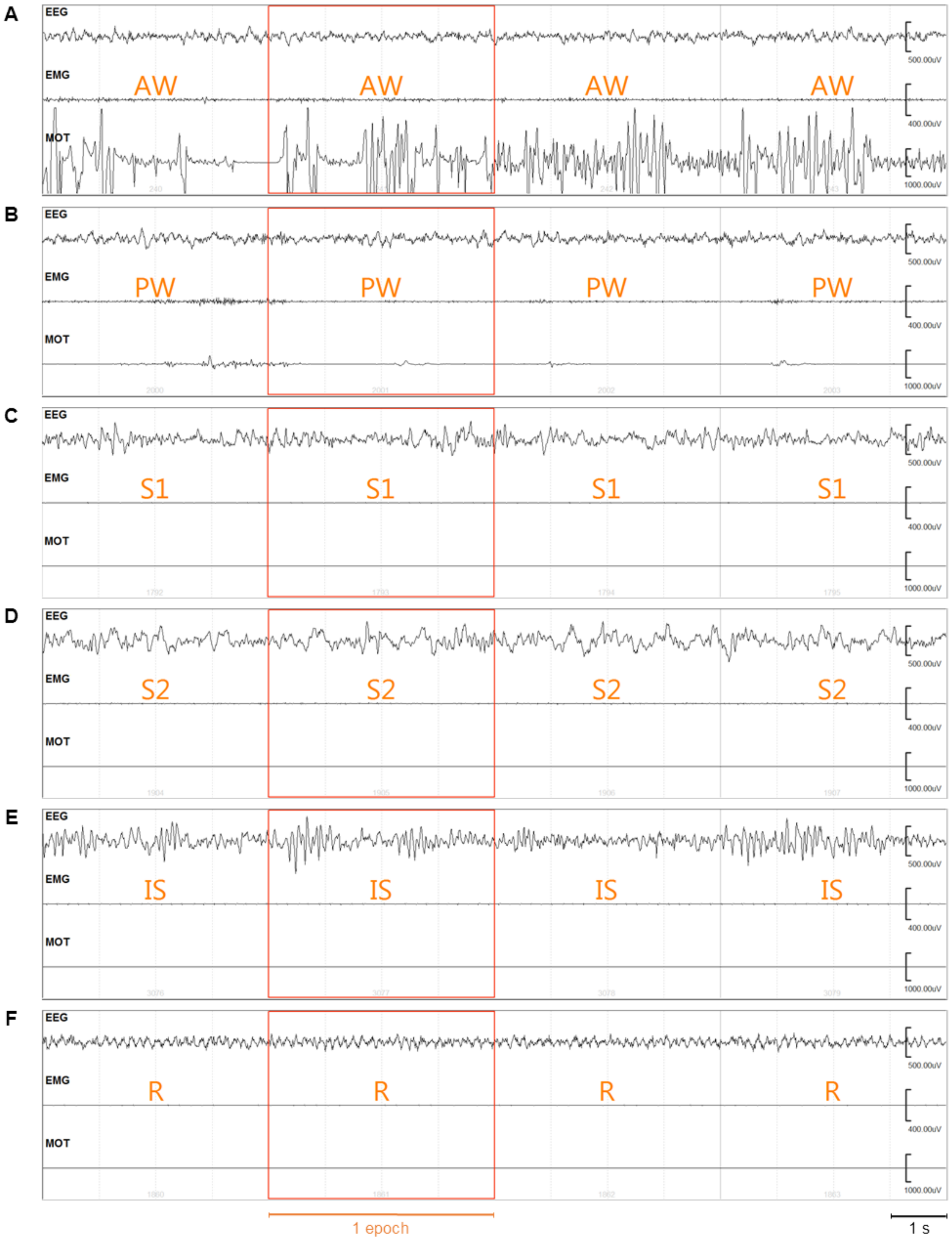

Supplementary Figure 1 - Representative electroencephalogram (EEG), electromyogram (EMG) and motility (MOT) traces in **(A)** active wakefulness (AW), **(B)** passive wakefulness (PW), **(C)** light slow-wave sleep (S1/SWS-1), **(D)** deep slow-wave sleep (S2/SWS-2), **(E)** intermediate stage of sleep (IS), and **(F)** rapid eye movement sleep (R/REMS).
